# Supplementary material for: Genome-Wide Identification, Evolution, and Expression Analyses of AP2/ERF Family Transcription Factors in Erianthus fulvus
Source: Int J Mol Sci. 2023 Apr 12;24(8):7102. doi: 10.3390/ijms24087102 (PMC10139229; doi:10.3390/ijms24087102)
Supplement: Supplementary file 1 [file ijms-24-07102-s001.zip › Supplementary Figures.pdf]

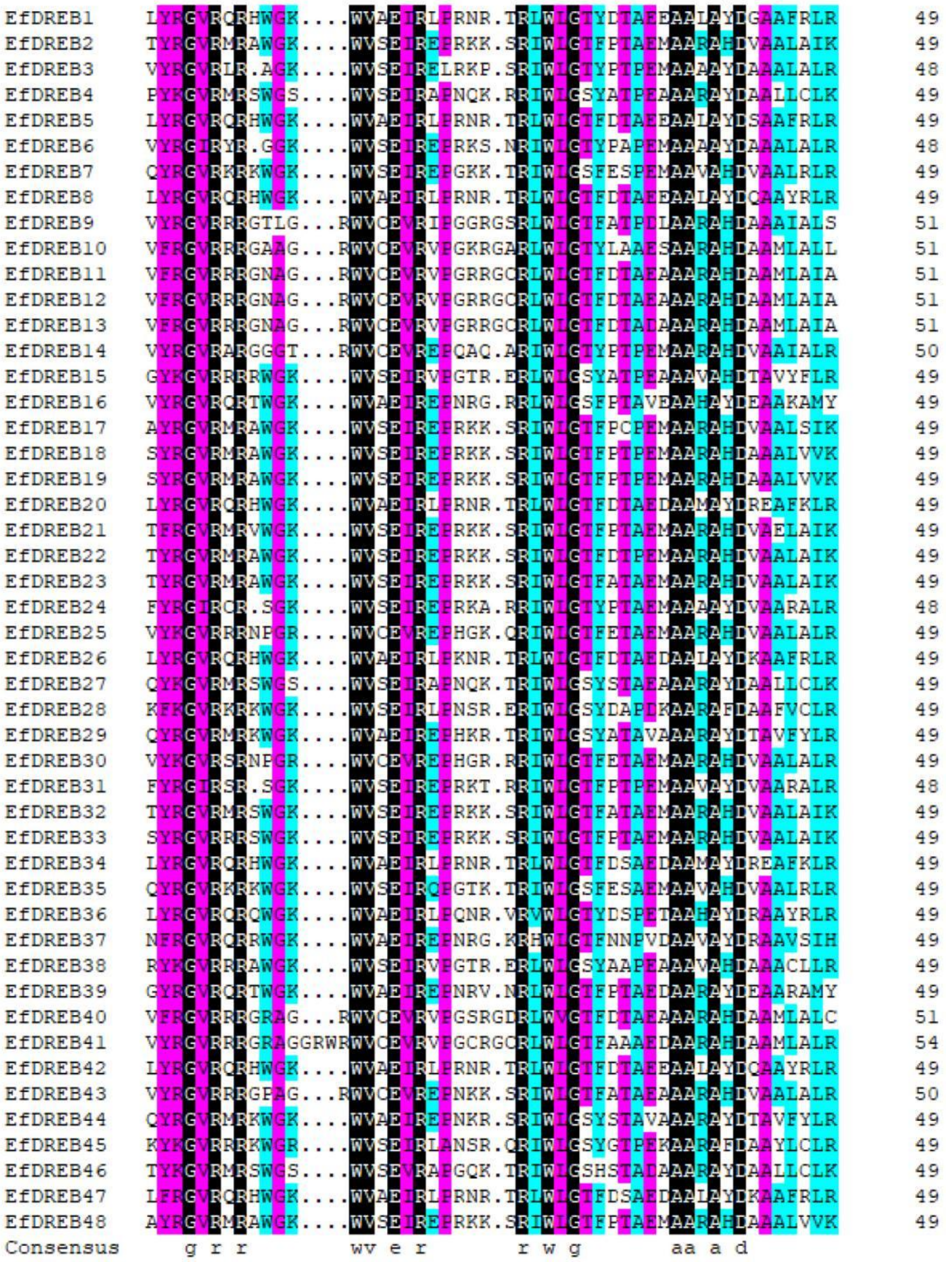

Figure S1. Multiple alignments of DREB subfamily proteins using the Clustal method by DNAMAN software. The conserved Val-14 (V) residue is important for distinguishing DREB family genes family genes.

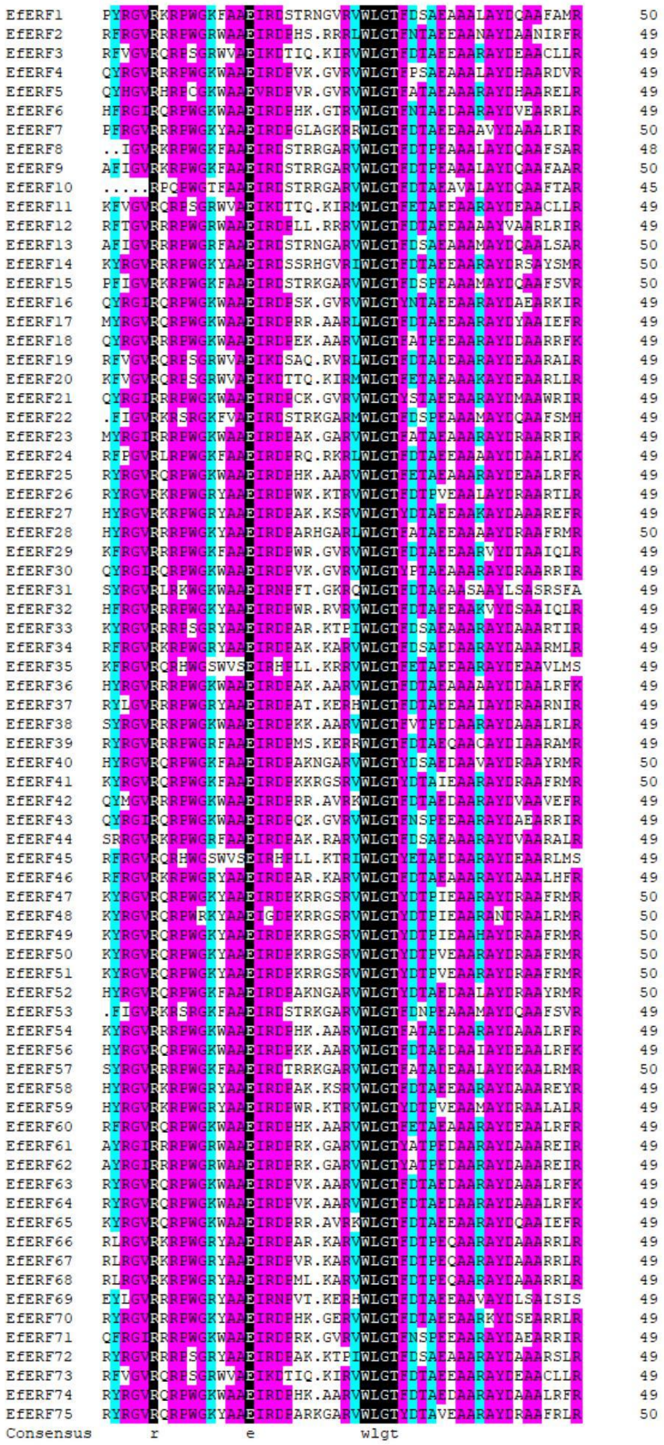

Figure S2. Multiple alignments of ERF subfamily proteins using the Clustal method by DNAMAN software. The conserved Ala-14 (A) residue is important for distinguishing ERF family genes. Although several genes did not contain an Ala-14 residue, they showed a close relationship by phylogenetic analysis and were thereby classified as ERFs.

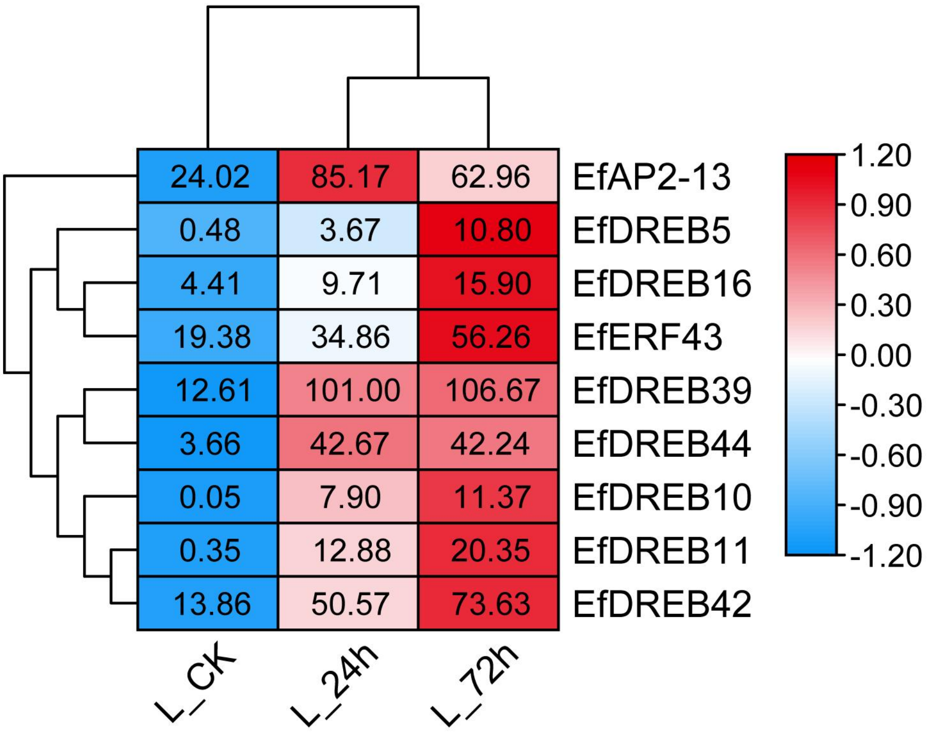

Figure S3. Expression profile of 9 *EfAP2/ERF* genes. Heatmap clusters of 9 *EfAP2/ERF* gene expression profiles in leaves (L) at the seedling stage under low-temperature (4°C) stress (0, 24 and 72 h). The heatmaps were created by TBtools based on the FPKM values. For each line, the expression patterns are presented as heatmaps in blue/white/red coding, with red indicating high expression level, white indicating moderate expression level, and blue indicating low expression level. Most notably, the expression levels of these 9 genes were much higher under cold stress than in the control.
